# Data on examining the role of human capital in the energy-growth nexus across countries

**DOI:** 10.1016/j.dib.2016.09.027

**Published:** 2016-09-23

**Authors:** Zheng Fang

**Affiliations:** School of Business, SIM University, Singapore

**Keywords:** Human capital, Penn World Table, Energy, Cross-country analysis

## Abstract

This article describes two publicly available data sources: the new generation of Penn World Table (www.ggdc.net/pwt) and the BP Statistical Review of World Energy (http://www.bp.com/statisticalreview) which can be used to examine the role of human capital in the energy-growth nexus across countries. The critical human capital measure across countries is for the first time made available in the Penn World Table 8.0 and it enables empirical researchers to conduct cross-country analysis involving human capital much easily than ever before.

**Specifications Table**

**Table:** 

Subject area	*Economics*
More specific subject area	*Energy Economics*
Type of data	*Links*
How data was acquired	*Publicly available online*
Data format	*Raw*
Experimental factors	*Publicly available data sources*
Experimental features	*The data are calculated based on/collected from government sources and published data.*
Data source location	*Global data*
Data accessibility	*Data is within this article.*
	*The Penn World Table is available at*www.ggdc.net/pwt
	*The BP Statistical Review of World Energy is available at*
	http://www.bp.com/statisticalreview

**Value of the data**

• The Penn World Table version 8.0 introduced human capital index.
• The Penn World Table contains key macroeconomic information for cross-country comparisons.
• The BP Statistical Review of World Energy provides energy reserves, production, consumption and prices by energy type and country for a long period of time.
• The Penn World Table and the BP Statistical Review of World Energy can be used for researches on energy-growth nexus which take into consideration human capital or trade.

## 1 Data

The data consists of real gross domestic product, physical capital, population, employment, human capital index from the Penn World Table (PWT) 9.0 and energy and electricity consumption from the BP Statistical Review of World Energy 2015 for 63 countries for a period 1970–2014. Real GDP and capital stock is in million 2011 US dollars, population and employment is in millions and human capital index is unit free. Energy and electricity consumption is in million tones oil equivalent and terawatt-hours respectively.

## 2 Experimental design, materials and methods

The data is retrieved from two well-known datasets – the Penn World Table and the BP Statistical Review of World Energy. There are a few major changes in the new generation of PWT [1]. First, the dataset introduced the measure of real GDP on the output-side that is more suitable for productive capacity comparison across countries than real GDP measure on the expenditure-side. Second, the dataset uses multiple International Comparisons Program benchmarks to keep the GDP measures more consistent over time. Third, human capital index and relative total factor productivity are included for the first time.

The human capital index available since PWT version 8.0 makes empirical researches with a focus on human capital and cross-country comparisons more viable [2]. It is calculated based on average years of education and an estimated rate of return to education from the Mincer equation [3]. That is,

$$\mathrm{hc}=e^{\phi (s)}$$

where

$$\phi (s)=\{\begin{array}{cc} 0.134s & \text{if}\;s\leq 4\\ 0.134*4+0.101(s-4) & \text{if}\;4<s\leq 8\\ 0.134*4+0.101*4+0.068(s-8) & \text{if}\;s>8 \end{array}$$

because the early years of education are evidenced to have a higher return than the later years in some studies [4] (although this assumption is still under debate).

In PWT 9.0, the average years of schooling is mainly from two sources, one is Barro and Lee [5] that covers 146 countries at five-year intervals from 1950–2010 and the other is Cohen and Leker [6] which includes data for 95 countries at ten-year intervals from 1960–2020. If only one source covers the country, the only source is used for the measure of years of schooling. If both sources cover the country, a cross-check using De La Fuente and Domenech [7] and/or the UNESCO data is conducted to determine the preferable source. If the sources for cross-checking are not available for specific countries, Barro and Lee data is used because it covers an earlier date back to 1950.

Energy data across countries can be obtained from the BP Statistical Review of World Energy [8]. The Statistical Review was first published in 1951 and all statistics are claimed to be taken from government sources and published data. It provides data on reserves, production, consumption and prices of traditional energies such as oil, gas and coal, as well as consumption of new energies such as nuclear energy, hydroelectricity, solar, wind, geothermal, biomass and other renewables. Primary energy consumption compiled in the excel file comprises commercially traded fuels including modern renewables that are used to generate electricity. Electricity data are only available from 1985 onwards.
