# Supplementary material for: Data on examining the role of human capital in the energy-growth nexus across countries
Source: Data Brief. 2016 Sep 23;9:540–2. doi: 10.1016/j.dib.2016.09.027 (PMC5054240; doi:10.1016/j.dib.2016.09.027)
Supplement: Supplementary file 1 — Supplementary material [file mmc1.docx]

Dear Editor,

This is to confirm that there is **no conflicts of interest** involved with the manuscript entitled “Data on examining the role of human capital in the energy-growth nexus across countries”.

Thank you very much for your consideration.

Best Regards,

Zheng Fang
